# Relationships between TGFβ Proteins and Oxygen Concentrations Inside the First Trimester Human Gestational Sac

**DOI:** 10.1371/journal.pone.0002302

**Published:** 2008-06-04

**Authors:** Shanthi Muttukrishna, Sangeeta Suri, Nigel Groome, Eric Jauniaux

**Affiliations:** 1 UCL EGA Institute for Women's Health, University College London, London, United Kingdom; 2 School of Biological and Molecular Sciences, Oxford Brookes University, Oxford, United Kingdom; AgroParisTech, France

## Abstract

In early pregnancy, the O_2_ gradient between the maternal circulation and the gestational sac tissues modulates trophoblast biological functions. The aim was to evaluate if placental partial pressure of oxygen (PaO_2_) modulates in vivo synthesis of specific placental proteins inside the first trimester gestational sac. Matched samples of peripheral venous blood, blood from the placental bed (PB), coelomic fluid (CF) and placental tissue were obtained in 37 normal pregnancies at 6–12 weeks gestation. PaO_2_ was measured in PB and CF using an IRMA blood gas monitor. Inhibin A, activin A, sEng, PlGF, sFlt-1 and free VEGF concentrations were measured in all samples. HSP 70 was measured in placental extracts. ANOVA showed ∼60% increase in PB PaO_2_ (P = 0.02) between after 10 weeks gestation. Unpaired Student's T-test between two groups (6–9 weeks vs 9–12 weeks) shows a significant increase in MS Activin A (P = 0.001), CF activin A (P<0.001), MS P1GF (P = 0.001), CF PlGF (P<0.001), MS sFLT-1 (P = 0.03), CF sFLT-1 (P = 0.01), HSP 70 in placental extracts (P = 0.04) and a significant decrease in PB inhibin A levels (P<0.001) and PB sFLT-1 (P = 0.02) . Multiple correlation analysis showed a significant negative correlation between PB inhibin A levels and gestation (r = −0.45, P<0.05) and PB PaO_2_ (r = −0.5, P = 0.008) and also between sFLT-1 and PB PaO_2_ (P = 0.03). There was a positive correlation (P<0.01) between PlGF, sEng and VEGF levels in the placental extracts. Our results indicate a direct relationship in the early intrauterine PaO_2_ in vivo and inhibin A and sFLT-1 concentrations confirming our hypothesis that specific placental proteins are regulated by intrauterine O_2_ tension.

## Introduction

**THE PLACENTA** in mammals is the essential interface between the maternal circulation carrying oxygen (O_2_)-rich blood and nutrients and the fetal circulation. In the past it has been assumed that the principal function of the organ is to supply the fetus with as much oxygen as possible, and to a large extent that is true in the latter half of pregnancy when fetal weight gain is greatest. Our combined *in vivo in vitro* investigations have resulted in a new understanding of the materno-fetal relationship during the first trimester of pregnancy and led to the hypothesis that the placenta limits, rather than facilitates, oxygen supply to the fetus during the period of organogenesis [1], [2]. The earliest stages of development therefore take place in a low oxygen environment, reflecting to some extent the evolutionary path [3].

The physiological hypoxia of the early gestational sac protects the developing fetus against the deleterious and teratogenic effects of oxygen free radicals but it is also necessary to maintain stem cells in a fully pluripotent state [4] for at physiological levels free radicals regulate a wide variety of molecules, in particular transcription factors [5]. In addition, it is now well-established that the first-trimester intra-uterine O_2_ gradient influences trophoblast proliferation and differentiation along the invasive pathway [6] and villous vasculogenesis [7].

There is increasing evidence that oxidative stress or an imbalance in the oxidant/antioxidant activity in utero-placental tissues plays a pivotal role in the development of placental-related diseases. Pre-eclampsia stems from a defect in early trophoblast invasion which may be sufficient to anchor the conceptus but is insufficient to fully convert the spiral arteries into low-resistance channels [8]–[10]. Incomplete conversion of the spiral arteries results in retention of smooth muscle cells within their walls which lead not only to diminished perfusion of the intervillous space, but more importantly to intermittent perfusion [11]. Since the placenta and fetus continually extract oxygen it is expected that transient hypoxia will result and that consequently the placenta suffers a chronic low grade ischaemia-reperfusion type injury [11], [12].

Only a few in vitro studies have investigated the role of vascular endothelial growth factor (VEGF) [13], nitric oxide, adhesion molecules [14], [15] and activin A [16], [17] on the regulation of trophoblast invasion. We have previously shown that inhibin A and activin A levels are higher maternal serum from 15 weeks gestation in pregnant women who subsequently developed pre-eclampsia [18]. A recent study has also shown that levels of sFLT-1 and sEng are elevated around 20 weeks gestation in patients who subsequently developed pre-eclampsia [19]. Inhibin, activin, VEGF, PlGF, sFLT-1 and Endoglin are members of the TGF β family. Soluble Endoglin is a placenta derived soluble TGF-beta co-receptor. VEGF is one of the most important factors regulating angiogenesis [20]. A recent study carried out in bovine aortic endothelial cells (BAEC) suggests that activin A induces capillary formation in these cells [21]. Placental growth factor potentiates the effect of VEGF and sFLT-1 antagonises the effect of VEGF. Soluble Endoglin inhibits the formation of blood vessels and induces vascular permeability and hypertension [22].

Increased oxidative stress in the placenta of women presenting with pre-eclampsia is well documented [3]. We hypothesise that changes in intrauterine PaO2 modulates placental protein secretion and synthesis as early as in the end of the first trimester of human pregnancy and could reflect a progressive defect in the normal placentation which is associated with the development of pre-eclampsia during the second half of pregnancy. To evaluate this hypothesis we have investigated ex vivo, the relationships between PaO_2_ and specific placental proteins inhibin A, activin A and pro and anti angiogenic factors PlGF, sFLT-1, sEng and VEGF concentration inside the early human gestational sac. Heat shock protein content (HSP 70) in the placental extracts was measured to study the effect of oxygen variation between gestations in early pregnancy.

## Materials and Methods

### Subjects and Samples

We have investigated a series of coelomic fluid (CF), blood from the decidua under the placental bed (PB) peripheral venous blood and villous tissue samples in 37 women undergoing surgical termination of pregnancy for psychosocial reasons at 6–12 weeks gestation. The study only included uncomplicated pregnancies and gestational age was determined from the first day of the last period and confirmed by ultrasound measurement of the fetal crown-rump length. Written consent was obtained from each woman after receiving complete information on the procedure. This study was approved by The University College London Hospitals Committee on the Ethics of Human Research.

In all cases, maternal peripheral venous blood was obtained from an antecubital vein prior to the surgical procedure. All patients received small dose (400–800 µg) of misoprostol 30–60 min before the procedure to prime the cervix. None of the women presented with any side effect and our previous studies have shown this medication does not have an effect on the samples collected [23]. CF and PB samples were all collected before the surgical termination of pregnancy, under general anaesthesia, by means of transvaginal needle aspiration under ultrasound guidance as previously described [23]. The blood samples were collected in pre-heparinized plastic gas-tight syringe. CF and PB O_2_ concentration (PaO2) was immediately measured on the IRMA blood gas analyser (IRMA True point blood analysis system, Edison, New Jersey, USA) within the operating theatre. The IRMA analyser was calibrated using a disposable calibration cartridge set-up at the temperature value obtained in vivo. Only six CF samples were collected in group 2 (9–12 weeks) as the Coelomic cavity becomes smaller with advancing gestation and the amniotic cavity expands. CF was snap frozen in liquid nitrogen. Maternal and the remainder of the placental blood samples were centrifuged at 3000 rpm for 10 minutes. Placental villous tissue was collected at the end of the surgical procedure, rinsed in sterile PBS and snap frozen in liquid nitrogen. Fluid, serum and tissue samples were all stored at −80°C until assayed.

### Methodology

#### Fluid and Serum Assays

Inhibin A and “total” activin A were measured in all biological fluids in duplicates using our in house ELISAs as described elsewhere [24], [25]. The intra and inter assay variation was <10%. The minimum detection limit for inhibin A was 2pg/ml and activin A was 50pg/ml.

Commercial ELISAs from R&D system (Abingdon, Oxford, UK) were used to measure PlGF, sEndoglin, sFLT-1, and “free” VEGF in fluids and serum samples according to the manufacturer's protocol. All samples were assayed in duplicates and the intra and inter assay variations were <12% for all assays. The minimum detection limit for the respective assays was PlGF: 3.9pg/ml, sEng: 62.5pg/ml, sFlt-1 31.3pg/ml and free VEGF 7.8pg/ml.

#### Placental protein extracts

Frozen placental tissue was homogenised and extracts were prepared as described elsewhere [26]. The placental extract total protein contents was estimated by a commercial Bradford protein assay from PIERCE (UK) using a BSA standard. The extracts were assayed for the specific proteins investigated in this study and normalised against the total protein content. HSP 70 (R&D systems, Oxfordshire, UK) was also measured in placental extracts in addition to the above proteins.

#### Statistical Analysis

All data were log transformed to obtain a normal distribution. Statistical analysis was carried out using the log transformed data. PB O2 was analysed in three separate groups using ANOVA. All collected samples measurements were analysed as two separate subgroups according to gestational age; early first trimester: 6–9 weeks (n = 16), and late first-trimester 9–12 weeks (n = 21). Unpaired Student's T-test was carried out to investigate the difference between the two groups of pregnant women. Pearson correlation was carried out to study the relationship between the measured parameters using SPSS statistical package (SPSS, Chicago Illinois, USA). Results were considered statistically significant at *p*<0.05. Graph pad prism (Graph Pad software inc, San Diego, California, USA) was used to do the normality test and to plot the graphs.

## Results

The median (range), gestational age (days) maternal age (years) and BMI in group 1 were; 55 days (42–63), 24 years (17–37) and 26.77 (19–32) respectively and in group 2 were; 72 days (64–96), 24.5 years (20–38) and 24.3 (18–29) respectively. Maternal age and BMI were not significantly different between the two subgroups.

### TGFβ proteins distribution in the different compartments

Significantly (P<0.001) higher concentrations of inhibin A and activin A in PB and in placental extracts compared to MS (table 1) confirms a placental origin of these proteins.

**Table 1:** Mean±SEM concentration of activin A, inhibin A, sEndoglin, PlGF, sFLT-1 and VEGF in the different gestational fluids in the first trimester (6–12 weeks).

	Coelomic fluid	Maternal Serum	Placental Blood	Placental Extract
Activin A (pg/ml)	1033.6±198	770.8±96.7	1655.4±282	1933±297
Inhibin A (pg/ml)	354.5±45.2	192.8±19.3	674.6±142	1540.7±61.7
sEndoglin (ng/ml)	41.26±0.7	72±2	89.7±4.3	59.7±7.3
PlGF (pg/ml)	14±2.26	25.11±5.11	208.9±27	58±9
sFLT-1 (ng/ml)	46.2±4	1.8±0.35	10.2±2.23	5.96±1.1
VEGF (pg/ml)	<10	<10	<10	12±2

High concentrations of sEng were measured in MS, PB and placental extracts. sEng levels were significantly (P<0.01) lower in CF than in the other compartments.

SFLT-1 was in significantly (P<0.001) higher concentrations in CF than in the other compartments whereas PlGF was significantly lower (P<0.001) in CF than in other compartments.

“Free” VEGF levels were below the detection limit of the assay in MS, CF and PB.

### Changes with gestational age

**Figure 1:**
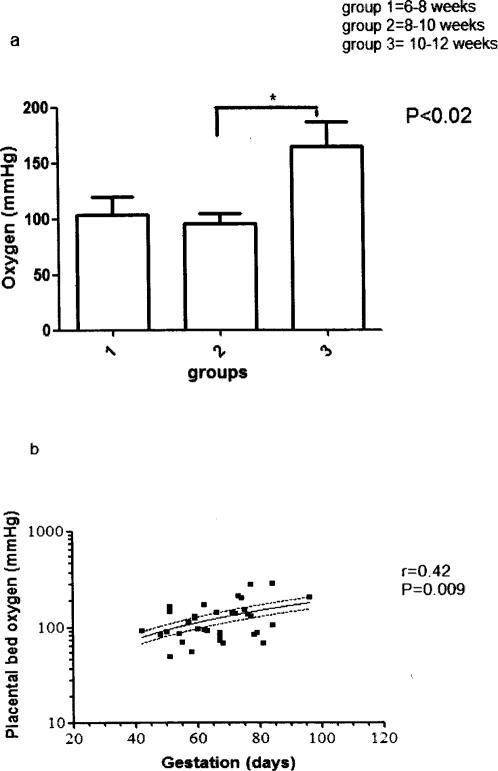
a: Placental bed oxygen concentration in early pregnancy. Mean±SEM values of placental bed blood concentrations in the different fluids measured. Group 1 = 6–8 weeks, group 2 = 8–10 weeks, group 3 = 10–12 weeks. P = 0.02, ANOVA. b: Relationship between gestation and oxygen concentration in the placental bed. Linear regression curve for PB PaO_2_ (log) vs gestation in days. 95% confidence interval lines are above and below the regression line. R = regression co-efficient and P = significance level. N = 37.

PB PaO_2_ significantly increased (∼60%, P<0.02, figure 1a) after 10 weeks gestation compared to PaO_2_ at 6–10 weeks gestation. Regression analysis showed a significant positive association (r = 0.42, P = 0.009) between PB PaO_2_ and gestational age (figure 1b).

**Figure 2:**
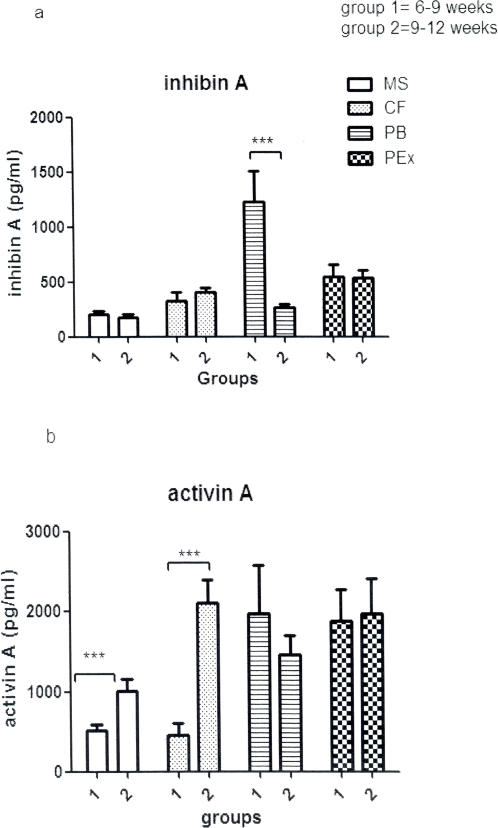
Inhibin A and Activin A in early pregnancy. Mean+SEM values of inhibin A and activin A concentrations in the different fluids measured. Group 1 = 6–9 weeks, group 2 = 9–12 weeks. *** = P<0.001, unpaired Student's T-test between the different groups.

Inhibin A in the PB significantly decreased (∼75%, P<0.001) from 6–9 weeks to 9–12 weeks (1227±282pg/ml, vs 270±30pg/ml). MS, CF and placental inhibin A concentration did not change with advancing gestation in the first trimester (fig 2a). Activin A levels in both the MS (∼2 fold, P<0.001, 522±78pg/ml to 1020±152pg/ml) and CF (∼4 fold, P<0.001, 455±162pg/ml to 2109±281pg/ml) increased significantly with gestation (fig 2b).

**Figure 3:**
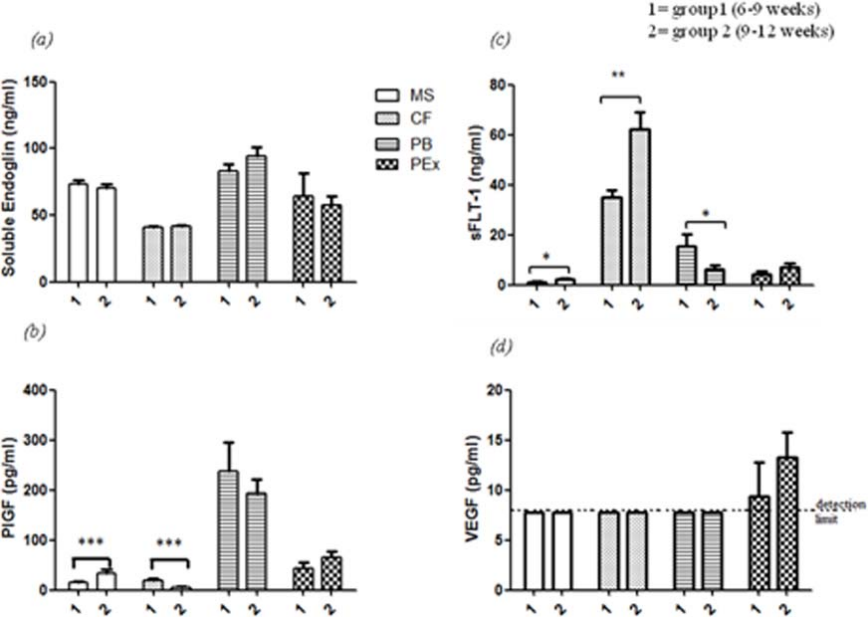
Pro and Anti angiogenic factors in early pregnancy. Mean+SEM values of soluble Endoglin, placental growth factor (PLGF), soluble VEGF receptor 1 (sFLT-1) and “free” vascular endothelial growth factor (VEGF) concentrations in the different fluids measured. Group 1 = 6–9 weeks, group 2 = 9–12 weeks *** = P<0.001, * = P<0.05 Unpaired Student's T-test between the different groups.

However, there was no difference in the levels of sEng between the two subgroups (figure 3a). P1GF levels in MS increased by >100% (15±2 vs. 32±9pg/ml, P = 0.001) between the two groups. P1GF levels did not change in the PB or placental tissue extract with advancing gestation. CF PlGF concentration only showed a significant decrease with advancing gestation (P<0.001, figure 3b).

Levels of sFLT-1 decreased significantly with advancing gestation in PB (16±5 vs 6.3±1.6ng/ml; P = 0.02) and an increase in MS ( P<0.03) and CF (P = 0.01, figure 3c). sFLT-1 levels did not change significantly in placental extracts between early and late first-trimester periods,

Placental extract contained very low concentrations of free VEGF and they did not change with advancing gestational age (figure 3d).

HSP 70 concentration in the placental extracts were significantly higher (P = 0.04) at 9–12 weeks (82.55+6ng/mg protein) compared to 6–9 weeks (51.3+5.7ng/mg protein) placentae.

### Multiple regression analysis

Table 2 shows all the significant relationships between the different molecules in the same compartment. An association between the same molecules in the different compartments is also shown in table 2. PB oxygen tension was negatively correlated to sFLT -1(r = −0.44, P<0.05) and inhibin A levels (r = −0.5, P = 0.01) in PB. PlGF, sEng and sFLT-1 were all significantly associated with each other in PB. In the MS Activin A was positively associated to PlGF and sEng.

**Table 2:** Pearson correlation analysis data showing significant relationships between the proteins and PaO_2_ concentration in the different compartments of the early gestational sac.

	Pearson Correlation
**Placental bed blood (PB)**	Activin A vs PlGF (r = 0.57, P<0.02) PlGF vs sFlt-1 (r = 0.57, P<0.01) PlGF vs sEng (r = 0.69, P = 0.001) sFlt-1 vs sEng (r = 0.42, P<0.05) sFlt-1 vs Po2 (r = −0.44, P<0.032)
**Maternal serum (MS)**	Activin A vs PlGF (r = 0.52, P = 0.005) Activin A vs sEng (r = 0.38, P = 0.05)
**Coelomic Fluid (CF)**	Inhibin A vs activin A (r = 0.6, P = 0.02)
**Placental Extracts (PEx)**	Inhibin A vs activin A (r = 0.73, P<0.001) VEGF vs PlGF (r = 0.67, P<0.001) VEGF vs sEng (r = 0.63, P<0.001) PlGF vs sEng (r = 0.54, P = 0.001)
**Inhibin A**	Gestation vs PB (r = −0.45, P = 0.02), PaO2 vs PB (r = −0.5, P = 0.01)
**Activin A**	CF vs MS (r = 0.8, P<0.001) CF vs gestation (r = 0.74, P<0.001) MS vs gestation (r = 0.54, P = 0.002)
**sFlt-1**	PaO2 vs PB (r = −0.44, P<0.05) CF vs gestation (r = 0.45, P<0.05)
**PlGF**	CF vs MS (r = −0.5, P = 0.025) MS vs gestation (r = 0.61, P<0.001)
**Endoglin**	MS vs PB (r = 0.56, P<0.01)

## Discussion

The results of the present study confirms that in vivo PaO_2_ in the uterine decidua under the placental bed (PB) increases after 10 weeks gestation as shown in our previous study [27] and suggest that the variations in the utero-placental O_2_ gradient during the first trimester of human pregnancy could have a major impact on the production of TGFβ proteins and thus modulate indirectly placentation and early foeto-placental angiogenesis in humans.

We found lower concentrations of sEng and PIGF and higher concentrations of s-Flt-1 in the exocoelomic cavity than in the other compartments whereas activin A and inhibin A concentrations were similar in CF and MS but lower than in PB and placental extract. Free VEGF was not detectable in CF samples. The exocoelomic cavity is the largest fluid space inside the first-trimester gestational sac [23]. It disappears at the end of the first trimester when the amniotic cavity reaches the chorionic plate of the placenta. Biochemical investigations of early fetal fluid have indicated that the CF results from an ultrafiltrate of MS and uterine gland secretions with the addition of specific placental and secondary yolk sac bio products [23]. The higher concentrations of human chorionic gonadotrophin (hCG), oestradiol, oestriol and progesterone found in the CF compared to MS indicate the presence of a direct pathway between the trophoblast and the early fetus [23].

VEGF and P1GF are vascular endothelial growth factors that play a key role in angiogenesis and vasculogenesis in a number of physiologic situations and in particular they play an important role during embryogenesis [20].The main source of VEGF molecules and P1GF during pregnancy is the placental trophoblast. sFlt-1 is a splice variant of VEGF receptor 1 (Flt1) and is produced by a variety of tissues including the placenta. SFlt-1 binds to both VEGF and PlGF in MS reducing their bioavailability [35]. Unbound PlGF, and VEGFs exist as free molecules and have a soluble receptor which facilitates its transport across membranes [35]. This could explain the higher total concentration of sFlt-1 in coelomic fluid.

Endoglin is a trans-membrane glycoprotein found on cell surfaces highly expressed in endothelial cells and syncytiotrophoblasts [36]. sEng is the soluble form of Endoglin found in serum. Its level is increased in the blood circulation of patients with angiogenic tumours, neovascularisation and myeloid malignancies and of pregnant women [22]. The presence of these pro and anti-angiogenic proteins in the CF in early pregnancy suggests that the exocoelomic cavity is also a reservoir for molecules with a direct role in the regulation of early fetal and placental angiogenesis.

Activin A and inhibin A are both glyco proteins, which have been identified in a wide variety of tissues including the placenta, adrenal glands, pituitary gland, bone marrow, kidneys, spinal cord and brain [37]. MS concentrations of activin A and inhibin A fall rapidly in the puerperium suggesting that the placenta is the primary source of these proteins throughout *most* of pregnancy. In the present study, the distribution of these proteins within the different compartments is consistent with this concept. It has been shown that inhibin and activin increase the production of GnRH and progesterone by the placenta, suggesting that they may play a role in the hormonal support of pregnancy [37]. It has also been shown that activin A promotes trophoblast invasion between 6–10 weeks in vitro [16]. Additionally, in bovine arterial cells, activin A has been shown to promote angiogenesis [21]. We found that in blood samples from the PB, activin A levels were positively related to PlGF, sFlt-1 and sEng whereas, in MS activin A levels were positively related to PlGF and sEng. These relationships suggest that activin A may also play a role in foeto-placental angiogenesis in early human pregnancy.

Anatomic and *in vivo* studies have shown that human placentation is in fact not truly haemochorial in early pregnancy [1], [3]. From the time of implantation, the extravillous trophoblast not only invades the uterine tissues but also forms a continuous shell at the level of the decidua and plugs in the tips of the utero-placental arteries. The shell and the plugs act like a labyrinthine interface that filters maternal blood, permitting a slow seepage of maternal plasma but no true blood flow into the intervillous space. This mechanical barrier results in intra placental PaO_2_ which are 2 to 3 times lower at 8–10 weeks than after 12 weeks. During pregnancy there is a progressive, but independent, increase in decidual PO_2_ advances between 7 and 16 weeks, which most probably reflects the increase in maternal blood flow volume within the uterine circulation. Biochemical analysis has also shown that the CF contributes to the redox potential of the gestational sac in a low oxygen environment [38] and in the fetal tissues antioxidant capacity at a time when the fetus is most vulnerable to oxidative stress [39].

Our data indicate a significant increase in HSP 70 expression in placental extracts between early and late first-trimester. In normal pregnancies, we have previously shown using immunohistochemistry that there is a physiological oxidative stress at around 9 weeks which is associated with an increased expression of heat shock proteins. This increased expression is particularly marked in the periphery of the placenta which is where the utero-placental circulation is first established [2], [3] SFlt-1 expression in placental villous explant from early first-trimester pregnancies (5–9 weeks) [28] and in vitro cytotrophoblast cell culture [40] is significantly increased under physiological low oxygen (<10%) conditions. In cytotrophoblast culture media, VEGF is not detected regardless of oxygen concentration whereas free PlGF is diminished by reduced oxygen (48). These results suggests that low oxygen levels play an important role in regulating TGF β proteins expression in the developing human placenta, however further interpretation of in vitro data is limited by laboratory experimental conditions. In our study, we found that in vivo placental bed serum inhibin A and sFlt-1 levels are inversely proportionate to the PaO_2_ measured in vivo at the same anatomical level. Recently it has been suggested that maternal endothelial cell dysfunction mediated by excess sFlt-1 is a major cause of the onset of the disease [22]. The inverse relationship that we have observed between increasing O_2_ concentration, inhibin A and sFLT-1 in blood samples from the PB could explain the increased concentrations of these proteins found in women presenting with pre-eclampsia [29]. Although sEng is a TGF beta receptor binding protein, the affinity of sEng to PlGF or VEGF is unknown.

In placental-related disorders of pregnancy such as pre-eclampsia, it has been reported that sFlt-1[18], [29], [30] and inhibin A serum levels are higher in patients weeks before they develop the clinical symptoms. Similar to the in vitro experiment at low O_2_ [40], PlGF levels have been reported to decrease in women presenting later in pregnancy with pre-eclampsia [30], [31], whilst sEng levels were found to increase [22], [32]. In addition, a recent systematic review has suggested that only in the third trimester sFLT-1 consistently increases and PlGF decreases in serum of women who subsequently develop pre-eclampsia, reaching a statistical significance in the early onset cases [33]. These results suggest that placental secretion of sFLT-1 could increase during the second trimester in women who subsequently present with clinical pre-eclampsia in the third trimester, long before these changes are detectable in maternal serum. These finding suggest that the molecules that are altered by PaO2 could be useful markers of the disease prior to the onset of the clinical symptoms as placental hypoxia in early pregnancy is one of the primary causes of the multisystem dysfunction later in pre-eclampsia.

In the present study, MS PlGF levels increased almost 2 fold from 6–9 weeks to 9–12 weeks gestation whereas sFlt-1 in the PB serum decreased by 75%. SFlt-1 has a high affinity binding to pro angiogenic proteins VEGF and PlGF [34]. VEGF was below the detection limit in CF, MS and PB samples measured in our study because the assay measures ‘free’ rather than ‘total’ VEGF. However, in placental extracts VEGF levels were above detection limit and it was positively correlated to PlGF and sEng levels. We also found that sFLT-1 levels in samples from the PB decreased with advancing gestation in the first trimester suggesting that as placental vascularisation increases with gestation, sFLT-1, an anti-angiogenic protein that controls the availability of pro angiogenic factors such as VEGF and PlGF are diminished contributing to an optimal development of the placenta vascularisation.

Our data indicate that inhibin A levels in samples from the PB decrease significantly (∼75%) between 6 to 12 weeks of gestation. These changes are similar to that reported previously in maternal serum for inhibin A and activin A between 8 to 12 weeks [25], [34]. Our correlation analysis showed that in samples from the PB, inhibin A levels were negatively correlated to PaO2. Inhibin A is also increased in the early second trimester in the serum of women who subsequently develop preeclampsia [17]. Furthermore, the significant inverse relationships found in samples from the PB between PaO2 and inhibin A and sFlt-1 concentration (table 2) suggest a probable mechanism for the rise in these molecules in placenta-related oxidative disorders of pregnancy. The observation of a similar trend in PB samples between PaO2, sFlt-1 and inhibin A suggests that these molecules could be used in combination for the screening of pre-eclampsia.

In conclusion this study showed an association between intrauterine oxygen tension and inhibin A and sFLT-1 confirming our hypothesis that oxygen tension may regulate placental protein secretion. The association between activin A, PlGF, sEng and sFLT-1 provides further evidence for activin A to be associated in angiogenesis.
